# Student performance in medical biochemistry and genetics: comparing campus-based versus zoom-based lecture delivery

**DOI:** 10.1186/s12909-022-03873-y

**Published:** 2022-11-16

**Authors:** Martha A. Faner, Raquel P. Ritchie, Katherine M. Ruger, Kirsten L. Waarala, Carol A. Wilkins

**Affiliations:** 1 College of Osteopathic Medicine, Detroit Medical Center, Michigan State University, Detroit, MI 48201 USA; 2 College of Osteopathic Medicine, Michigan State University, Macomb University Center, Clinton Twp, MI 48038 USA; 3 College of Osteopathic Medicine, Michigan State University, East Lansing, MI 48824 USA

**Keywords:** Assessment outcomes, In-person instruction, Online instruction, Undergraduate medical education

## Abstract

**Background:**

We appraised the effectiveness of online (Zoom) delivery versus live campus-based delivery of lectures in biochemistry and genetics courses and assessed the security of remote versus campus-administered exams.

**Methods:**

Participants were 601 students entering Michigan State University College of Osteopathic Medicine in 2019 or 2020. The former cohort completed courses and exams on campus, while the latter completed courses online due to the COVID-19 pandemic. For the biochemistry and genetics courses, the same lecturers delivered the same content and used identical exam questions for assessments in 2019 and 2020. The investigators compared percent correct for each question in 2019 and 2020.

**Results:**

This study found 84 of 126 (67%) of the questions yielded little difference (3% or less in % correct) between live delivery and Zoom delivery. For questions whose % correct index differed by 4% or more, Zoom delivery yielded a better performance for 16 questions (13%), while 19 questions (15%) showed live lectures performed better. Seven of the questions (6%) had an identical mode of delivery in 2019 and 2020 (e.g., self-study exercise). These served as “control questions” for which equivalent student performance was expected. The 126 questions analyzed spanned a wide range in the % correct index, from 60% correct to > 90% correct.

**Conclusions:**

The results suggest that Zoom and on-campus delivery of the content in biochemistry and genetics yielded similar achievement of course objectives. The high concordance, between 2019 and 2020, of the % correct for individual questions also speaks to exam security including online proctoring.

**Supplementary Information:**

The online version contains supplementary material available at 10.1186/s12909-022-03873-y.

## Background

The COVID-19 pandemic and the ensuing requirements for social distancing have provoked a sea-change in medical education in the pre-clerkship years: transition to remote learning (online lectures and small-group interactions), modifying hands-on clinical instruction, and adaptation of testing environments for assessment. A plethora of ideas and description of actual practices have been put forth to convert curricula to online learning and administer exams remotely [1, 2]. There are descriptions, for example, on a “Quarantine Curriculum” [3], a student-led development of a “COVID-19 Curriculum” [4], as well as discussions on the role of medical students’ government [5] and opportunities to make changes to the assessment of outcomes [6]. Some consequences of these changes, necessitated by the pandemic, have recently been analyzed and reported, mostly in terms of surveys regarding subjective feelings and impressions regarding the pandemic’s effect on student learning and outcomes [7–9]. Our study, however, used individual assessment items to measure student performance outcomes based on modes of content delivery used prior to and during the pandemic.

When COVID-19 cases emerged in Michigan during the spring of 2020, Michigan State University College of Osteopathic Medicine (MSUCOM) changed its campus-based lecture delivery to virtual delivery of lectures via Zoom webinars or meetings. The instructional team for two first-year courses in biochemistry and molecular biology (BMB) recognized the unique opportunity to compare the effectiveness of the two modes of content delivery: campus-based versus online. The team made a conscious decision to maintain the same lecturers delivering the same content and used identical exam questions to assess that content so that student performance on individual assessment items could be compared across cohorts. While the transition to online instruction and assessment was a necessity due to the pandemic, there may be positive aspects of online learning to consider and perhaps adopt to some extent even after the pandemic [10]. The researchers wished to determine whether online learning was as effective as methods of delivery traditionally used in our program.

Prior studies on comparing on-campus versus virtual/online delivery of content have yielded mixed results. Several studies tout superior outcomes using e-learning [11–13]. Other studies indicate traditional campus-based classrooms yield better assessment outcomes [14, 15]. Still other studies suggest there is no difference in the learning outcomes based the two learning formats [16, 17]. In the present paper, we report the analysis of first-year medical students’ performance in terms of the degree of difficulty of individual assessment items. The comparison was made to determine the effect, if any, of Zoom-based delivery of lectures in the 2020 cohort relative to campus-based delivery of the same content in 2019. The data and analysis may be of interest to students, faculty, and administrators who experienced the pandemic as well as those involved in post pandemic curriculum development regarding the value of incorporating online learning modalities.

## Methods

### Subjects and inclusion criteria

This study compared the exam performance of first-year osteopathic medical students enrolled at MSUCOM in biochemistry (BMB 516; Semester 1) and in molecular biology and genetics (BMB 528; Semester 2) at MSUCOM. The comparison was made to determine the effect, if any, of virtual (Zoom-based) delivery of lectures necessitated by the COVID-19 pandemic in 2020 for the Class of 2024, relative to campus-based lecture delivery in 2019 for the Class of 2023. Each year, the entering class consisted of approximately 300 students, with approximately 200 located at the East Lansing (EL) site, 50 at Detroit Medical Center (DMC) site, and 50 at Macomb University Center (MUC). All three sites follow a unified curriculum. The inclusion criterion was osteopathic medical students with enrollment and completion of the two BMB courses in either 2019 or 2020, resulting in a sample size of 301 in 2019 and 300 in 2020.

### Ethics approval

This study was a retrospective analysis of student grades and admissions data. The data was deidentified and used in aggregate. All of the data is associated with standard educational activities in the College of Osteopathic Medicine. The Michigan State University Institutional Review Board on Human Subjects has approved this study (IRB number 00005873).

### Instrument of measurement

In 2019, we delivered lectures in on-campus classrooms; in 2020, we delivered them virtually via Zoom webinars (see Lecture Delivery, below). The same lecturers delivered the same content with very minor exceptions, and we used identical exam questions to assess the same content. For each question, we compared the % correct in the item analysis report of ExamSoft (see Examinations, below). The % correct parameter is calculated as the number of examinees who answered an item correctly divided by the total number of respondents for that item multiplied by 100. This statistic provides an indicator of the degree of difficulty of the question, where the lower the value of % Correct, the more difficult the question was for the students.

Item difficulty is relevant for determining whether students have learned the concept being tested. It also plays an important role in the ability of an item to discriminate between students who know the tested material and those who do not [18]. The mathematical basis for this conclusion has been analyzed by Lord [19].

There were 159 questions administered in six exams within the BMB 516 and BMB 528 courses (last column, Table 1). Some of the questions in each examination did not lend themselves to a direct comparison, either because of a slight change in wording necessitated from student feedback in 2019 or because the mode of delivery did not involve lectures. These questions were grouped under “Other” (Column 6) in Table 1. We compared student performance in 2019 versus 2020 for 126 questions, accounting for ~ 80% of the examination questions used in both years (“Number Compared”, Table 1).

**Table 1:** Classification and number of questions: 2019 versus 2020 exams^a^

Exam	Category I (control questions)	Category II 3% or less difference	Category III 4% or more 2019 < 2020	Category IV 4% or more 2019 > 2020	Other	Number Compared	Total Number
BMB 516, Exam 1	1	15	3	4	10	23	33
BMB 516, Exam 2	0	19	4	4	6	27	33
BMB 528, Exam 1	0	16	3	2	0	21	21
BMB 528, Exam 2	2	14	1	3	4	20	24
BMB 528, Exam 3	3	12	1	3	5	19	24
BMB 528, Exam 4	1	8	4	3	8	16	24
Total	7	84	16	19	33	126	159

Category I --- The mode of delivery of content examined in these questions was identical in 2019 and 2020 (e.g., via assigned reading material or self-study exercise). These served as our Control Questions

Category II --- There was a difference of 3% or less in % Correct between 2019 and 2020

Category III --- There was a difference of 4% or more in % Correct, with 2020 Zoom delivery showing a better performance or higher % Correct than 2019 on-campus delivery (2019 < 2020)

Category IV --- There was a difference of 4% or more in % Correct, with 2019 on-campus delivery showing a better performance or higher % Correct than 2020 Zoom delivery (2019 > 2020)

Other --- Questions for which performance in the 2 years cannot be easily compared, either because of a change in the wording of a particular question or because the mode of content delivery was altered (e.g., a self-study exercise in 2019 was replaced by a lecture in 2020)

^a^In Categories I – IV, identical questions were used to assess the same content

For seven of the questions we analyzed, the mode of delivery of content was identical in 2019 and 2020 (e.g., assigned reading or self-study exercise); these served as our “control questions” (Category I, Table 1) for which we expected student performance to be the same from year to year. Indeed, the % Correct was within 3% of each other for six of these questions. One question yielded 57% Correct in 2019 versus 68% Correct in 2020, representing the lone exception. The standard error of the mean for % Correct of all 14 control questions (seven from each year) was 3.63. Since the standard error of the mean represents a measure of the dispersion of a sample around the population mean (e.g., how much variation would be expected by random sampling of the population), we used 3% difference as the criterion in the groupings described in Categories II-IV and reported under Results.

### Examinations

During the first two semesters at MSUCOM, students are assessed at regular intervals (2–4 weeks) and each exam contained assessment items from more than one course; the number of questions in an exam reflected the number of sessions of a particular course during that period. In the two BMB courses, individual questions were generated by faculty members and tagged with instructional objectives. After vetting by the course instructional team, the Course Coordinator enters the questions into the secure assessment platform ExamSoft (https://examsoft.com/). The Semester Director then assembles an exam from the question pool, and it is vetted a second time by the Course Coordinators that have content on that exam.

ExamSoft allows for secure computer-based testing. In 2019, the exams were conducted in on-campus classrooms with identification verification, in-person proctoring and other security measures (checking pockets etc.). In 2020, the exams were conducted remotely using ExamMonitor for virtual proctoring. ExamSoft reports an item analysis of the questions used in a particular examination. In this report, we focused on the “% Correct” parameter (as mentioned previously) as an indicator of the degree of difficulty of the question; the lower the value of % Correct, the more difficult the question was for the students.

### Statistical analysis of the data

Class averages, standard deviations, and standard error of the mean were calculated using the Microsoft Excel spreadsheet software. Chi-square analyses were carried out using an online statistical analysis program (https://www.socscistatistics.com/tests/chisquare2/default2.aspx).

### Lecture delivery

In both years, Semester 1 spanned 9 weeks from June 15 through August 14; BMB 516 was a 1-credit course (a combination of approximately 12 lectures and 5 activity sessions), taught alongside an 8-credit anatomy course and a 1-credit epidemiology and biostatistics course. In both years, BMB 528 (2-credit course consisting of a combination of approximately 29 lectures and 3 activity sessions) spanned the first 8 weeks of Semester 2, during which pathophysiology, immunology, and microbiology courses were also ongoing.

In 2019, the mode of lecture delivery was in campus-based classrooms. Each live lecture had a point of origination (e.g., DMC) in which students were physically in the classroom with the lecturer (see Additional file 1). At the non-origination sites (e.g., EL and MUC), students received the lecture via Polycom video conferencing in real time. There was a faculty member in the non-origination site classrooms to direct questions or discussion to the lecturer or to whom students could interact with following the session. It should be emphasized that, despite being “remote from the lecturer,” students at non-origination sites were physically in the classroom with their peers, along with a content expert faculty. More importantly, both internal course grades (see Additional file 2) as well as external metrics such as performance on national licensure exams (COMLEX-USA Level 1) [20] indicate no statistically significant differences in outcome measures between site-of-origin and receiving sites.

In 2020, the COVID-19 pandemic necessitated the switch to lecture delivery via Zoom webinars. To the extent possible, we maintained the same lecturers delivering the same content with very minor exceptions. In addition to the lecturer, we staffed each webinar (lecture session) with another faculty member to bring any major point of misunderstanding related to content to the attention of the lecturer, assist with any technical issues, and triage questions from students submitted through the Q&A function of Zoom, to be addressed at appropriate junctures during the session.

### Class attendance and access to lecture recordings

Since 2014, the instructional team in BMB has been engaged in a project to increase class attendance and engagement by rewarding real-time responses to i > Clicker questions during class sessions. Each lecture had 2–4 i > Clicker questions related to the lecture’s content. Students must answer at least one question correctly to qualify for the points, which contribute up to 2.4% of the points counting toward the final course grade. The number of students responding to i > Clicker questions was used to track attendance for BMB 528 in 2019. Zoom webinar attendance for BMB 516 and BMB 528 in 2020 was tracked by the unique views in the Zoom Attendee Report which was pulled out after the lecture.

The lectures in the BMB courses were recorded and posted to a repository for asynchronous access. In 2019, the recordings were captured during the Polycom broadcast between the three sites; in 2020, the recordings were captured during the Zoom webinar. In conjunction with the i > Clicker project, we also monitored the frequency that the recording of a particular lecture was being accessed for viewing.

## Results

### Item analysis of examination questions in 2019 and 2020

**Fig. 1:**
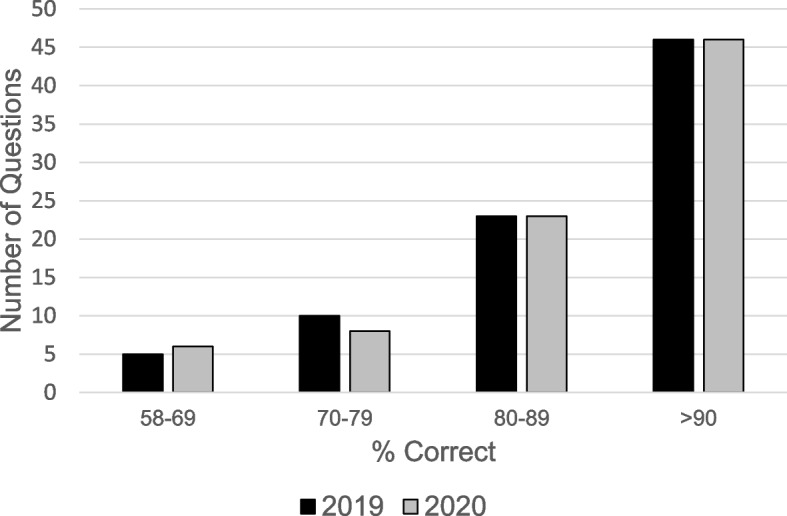
Comparison of the histograms, 2019 (black bars) versus 2020 (gray bars), showing the number of Category II questions that yielded % Correct in the ranges 58–69, 70–79, 80–89, and > 90. For each of the questions in Category II, there was a difference of 3% or less in the % Correct index between 2019 and 2020. The two distributions were not significantly different (*p* = 0.9534)

We found 84/126 (~ 67%) of the questions showed little/no difference (a difference of 3% or less in % Correct) between live campus-based delivery (2019) and Zoom delivery (2020). These were grouped into Category II in Table 1. The questions in Category II constitute a majority of the questions in any exam, across all six exams in the two courses and therefore, spanned the entire range of scientific content of the two courses (Table 1). Moreover, the questions encompass, in terms of Bloom’s taxonomy, both the recall/remembering type as well as questions of higher levels of cognition involving understanding, analysis, and application. The questions in this category span a wide range in terms of the % Correct index (Fig. 1), with ~ 45% of them in the < 90% Correct range. The histogram distributions for 2019 and 2020 look very similar; this conclusion is supported by a chi-square analysis, which showed that the two distributions were not significantly different (*p* = 0.9534).

We divided those questions in which the % Correct index differed by 4% or more between 2019 and 2020 into two categories (Table 1). There were 16 questions (~ 13%) that showed better performance in 2020 than in 2019. In other words, a Zoom delivery yielded a higher % Correct in each of the questions in Category III. On the other hand, there were 19 questions (~ 15%) that showed better performance in 2019 than in 2020. In other words, campus-based delivery yielded a higher % Correct in these questions in Category IV. The number of questions in Categories III and IV essentially balance each other out across all six exams and therefore, the delivery method (campus-based versus Zoom-based) did not differentially impact any particular topic of scientific content. Together with the large fraction of questions in Category II, showing little/no difference between 2019 and 2020, these results suggest that conversion of lecture delivery from campus-based classrooms to Zoom webinars online did not significantly affect student learning and performance.

### Class characteristics and student performance, 2019 versus 2020

The first-year students entering MSUCOM in 2019 (Class of 2023) and in 2020 (Class of 2024) presented with equivalent academic credentials (Table 2). Both the overall undergraduate GPA as well as the GPA in science courses were within a standard deviation of each other. Similarly, there was no significant difference in terms of the composite MCAT scores and in terms of specific MCAT categories such as Critical Analysis and Reasoning Skills.

**Table 2:** Admissions data for the Classes of 2023 and 2024 at MSU COM

	Class of 2023 (2019)	Class of 2024 (2020)
Number of students	301	300
Mean GPA^a^ ± STDEV^b^	3.63 ± 0.26	3.66 ± 0.24
Mean Science GPA ± STDEV	3.58 ± 0.29	3.61 ± 0.26
Mean MCAT^c^ ± STDEV	505 ± 4	506 ± 4
MCAT – CARS^d^ ± STDEV	125 ± 2	125 ± 2
MCAT – CPBS^e^ ± STDEV	126 ± 2	126 ± 2
MCAT – BBFL^f^ ± STDEV	127 ± 2	126 ± 2

^a^*GPA* grade point average earned for the undergraduate degree

^b^ ± *STDEV* standard deviation of the mean

^c^*MCAT* Medical College Admission Test

^d^*CARS* Critical Analysis and Reasoning Skills

^e^*CPBS* Chemical and Physical foundations of Biological Systems

^f^*BBFL* Biological and Biochemical Foundations of Living systems

Consistent with these data on entry into medical school, the two classes performed comparably in the early exams of their respective first two semesters (Table 3). Two key considerations regarding exams and the grading system at MSUCOM may be important in terms of this table: (a) Each exam contained assessment items from concurrent courses during the semester; the number of questions in an exam reflected the number of sessions of a particular course during that period. (b) MSUCOM used a P/N (pass/no-pass) grading system with 70% as the minimum required for passing the BMB courses. When considering questions derived from the BMB course only, campus-based delivery in 2019 yielded a slightly higher class average for five of the six exams compared to Zoom delivery in 2020, although the class averages for each of the corresponding exams in the 2 years were within 3 percentage points of each other (Table 3, Part A). This is counterbalanced by the observation that, in terms of the overall exam covering all content, the class averages were higher in 2020 (Zoom-based delivery) than in 2019 (campus-based delivery) (Table 3, Part B). It is possible that, in the P/N grading system, students felt comfortable in terms of the number of points they had in the BMB course such that more study or attention was devoted to the content of the other concurrent course(s).

**Table 3:** Summary of the class averages on exams: BMB and all content questions

Exam	2019	2020
**A) BMB Content Only**^**a**^	Class average (%) ± STDEV	Class average (%) ± STDEV
BMB 516, Exam 1	81 ± 11	84 ± 11
BMB 516, Exam 2	87 ± 8	85 ± 9
BMB 528, Exam 1	81 ± 10	80 ± 10
BMB 528, Exam 2	86 ± 9	85 ± 8
BMB 528, Exam 3	86 ± 9	84 ± 9
BMB 528, Exam 4	82 ± 10	81 ± 10
**B) All Content**^**b**^	Class average (%) ± STDEV	Class average (%) ± STDEV
Semester 1, Exam 1	78 ± 10	84 ± 9
Semester 1, Exam 2	83 ± 7	86 ± 7
Semester 2, Exam 1	85 ± 8	84 ± 9
Semester 2, Exam 2	84 ± 8	85 ± 7
Semester 2, Exam 3	84 ± 8	87 ± 8
Semester 2, Exam 4	83 ± 8	84 ± 8

^a^Each exam contained assessment items from more than one course; part A analyzed questions from the BMB course. The data are expressed as class average (in %) ± standard deviation (STDEV)

^b^Part B provides the class average covering all questions in a particular exam, one part of which contained the BMB questions analyzed in part A. The data are expressed as class average (in %) for the entire exam ± standard deviation (STDEV)

**Fig. 2:**
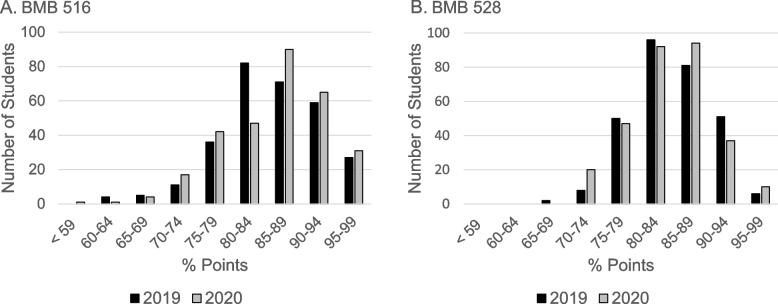
Comparison of the histograms, 2019 (black bars) versus 2020 (grey bars), showing the number of students who earned the percentage of points counting toward the final course grade in the ranges < 59 through 95–99, in intervals of 5%. **A** BMB 516; **B** BMB 528. In both courses of each year, a P-grade (passing) required 70% of the total number of points in the course

This notion is supported by comparing the histogram distribution of the number of students in a particular interval of the percent of total points counting toward the final grade (Fig. 2). For both BMB courses, there were more students in the 80–84% range in 2019 while, in 2020, there were more students in the 85–89% range. As shown in Fig. 2, the number of N-grades in BMB 516 was 9 in 2019 while the corresponding number in 2020 was 6; the number of N-grades in BMB 528 was 2 in 2019 while the corresponding number in 2020 was zero. Coupled with the item analysis of exam questions, these results on student performance suggest that online Zoom delivery of the content in biochemistry and genetics yielded as good, if not better, an outcome as on-campus classroom delivery.

### Lecture attendance and access to lecture recordings, 2019 versus 2020

Class attendance averaged between ~ 80% to ~ 90% in a course-specific and year-specific manner (data not shown). Attendance in BMB 516 averaged ~ 91% in 2020 but dropped off in the second semester to 83% for BMB 528. In 2019, the same BMB 528 course (second semester for Class of 2023) averaged 91% in attendance, most likely incentivized to a large extent by the possibility of earning “clicker points” (described under Class Attendance and Access to Lecture Recordings in the Methods section). This difference in attendance may be reflected, in all four exams in the course, by the slightly higher class averages in 2019 than the corresponding scores in 2020.

**Fig. 3:**
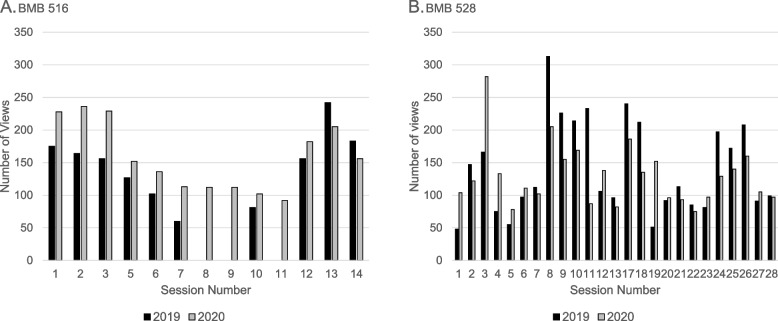
Comparison of the histograms showing the number of views of lecture recordings in 2019 (black bars) versus 2020 (gray bars). **A** BMB 516; **B** BMB 528

We have also monitored the frequency of asynchronous student access to lecture recordings (Fig. 3). For BMB 516 in the first semester, student access was higher in 2020 than in 2019. For BMB 528 in the second semester, the frequency of access to the recordings was reversed, with more students accessing recordings in 2019 than in 2020.

## Discussion

This study compared the performance, for each examination question in biochemistry and genetics, the % Correct index yielded by students who learned the content either via online Zoom delivery in 2020 or via on-campus classroom instruction in 2019. For most of the exam items analyzed, those questions with a low % Correct (high degree of difficulty) in 2019 yielded a very similar low % Correct index in 2020 while questions with a high % Correct (low degree of difficulty) in 2019 also resulted in a high % Correct index in 2020.

The high concordance, between 2019 and 2020, of the % Correct index for individual questions speaks to the security of the exam questions in the ExamSoft bank. In addition, the data also lend some confidence to the ExamMonitor remote proctoring process. Most important of all, however, we believe that the data provides the basis for being especially proud of the performance and resilience of our medical students. Together, the data and analysis reported here may provide some level of comfort to students, faculty, and academic administrators in terms of achievement of instructional objectives and integrity of scholarship during a difficult time.

There are several recent reports on the effect of the pandemic on the teaching of the foundational sciences in medical education. For example, Shahrivini et al. [7] reported a survey of first- and second- year medical students regarding the impact of remote learning on quality of instruction and ability to participate and on preparedness for subsequent stages of medical training. While students preferred the flexibility of learning at their own pace, many students felt isolated and less-than-connected to their classmates and medical school. On the other hand, Suneja et al [8] reported based on final scores of assessments, the learning outcomes were fully attained in terms of the first-year curriculum including anatomy, physiology, and biochemistry along with professional and personal development modules. In fact, the disruption of physical classrooms, necessitated by the pandemic, led to unanticipated benefits derived from increased use of technology, at both the faculty and student levels. Our study complements and supplements these reports by providing a direct item-by-item comparison of individual examination questions.

We wish to discuss a point of difference between our study, compared to other studies of online learning, that exists due to the nature of our curriculum. Reviews of the distinguishing features of online versus campus-based delivery of content often emphasize the “asynchronous and flexible nature” of the online medium [7, 21, 22]. Three limitations of the present study need to be brought out in this context. First, each of the lectures in our study, in both years, were scheduled for a specific date and time. Therefore, to interact with the lecturer, a student had to attend the lecture physically in a campus classroom (2019) or attend the Zoom webinar in real time (2020). In this sense, the lectures in both years were synchronous. Second, students had access, asynchronously, to recordings of the lectures in both years. Finally, in our pre-COVID instructional model, MSUCOM had three sites connected by video conferencing during a lecture. A student at a non-origination site might attend the lecture via a connected classroom; alternatively, a student could also live stream the lecture from a remote location. It is important to note that Hortos et al. used external metrics, such as performance on national licensure exams (COMLEX-USA Level 1), to show that there were no statistically significant differences in outcome measures between site-of-origin and receiving sites [18]. Moreover, we have also found that there has been no significant difference in average course grades for the BMB courses between the three sites going back to 2012 (see Additional file 2 for 2018 and 2019 and data not shown for previous years), further supporting that there were no statistically significant differences in outcome measures between site-of-origin and receiving sites. In this connection, it should also be noted that in post-course evaluations in 2020, ~ 58% of the students indicated a preference for synchronous live webinars while approximately 33% preferred asynchronous pre-recorded lectures. Despite the flexibility afforded by a pre-recorded lecture, the students wanted a scheduled event, noting that they would have access to the recording in either case. One interpretation is that these students longed for direct interactions with the faculty and their peers because they had not been able to congregate physically as a class in medical school.

In addition to the point discussed above, two other possible limitations need to be brought out regarding our study. First, we recognize the difficulty in determining student engagement during lecture delivery via Zoom, particularly since the camera of each student was not turned on at any time during the session. However, even during in-person lectures, there are challenges in determining student engagement; they could be physically present in the classroom but engaged in other activities (e.g., social media or online shopping). Second, we also acknowledge that there are other aspects of in-person learning that support medical students and develop valuable skills besides the mere acquisition of biochemistry knowledge and our study does not address those skills. Another study at MSUCOM was designed to gauge student perceptions of the effect of COVID-19 on areas such as communication, wellness, and curriculum. This survey was administered to the pre-clerkship students at MSUCOM in the spring of 2020 to better understand how changes due to COVID-19 affected them. This study is beyond the scope of this current paper and the data will be discussed in a subsequent publication.

Even before the pandemic, there were calls for significant changes in medical education, with suggestions to overcome barriers to online learning [10] and with predictions that pre-clerkship curriculum will be delivered completely online [23]. Online learning can increase the accessibility of medical education since it could be available almost anywhere and anytime. For pre-clerkship medical curriculum, it is easy to imagine a blended model where students attend some sessions in person while others are available for the learner to complete at their own pace. This is a more student-centered approach and allows more flexibility to pursue research, community engagement and self-care. All these positive aspects of online learning argue that it should be adopted to some extent even after the pandemic.

## Conclusions

While many studies comparing online versus campus-based learning carried out during the pandemic used surveys to measure subjective feelings and impressions of student learning, our study measured student performance outcomes. As a result, our present report contributes to the discussion regarding the value of increased use of online learning modalities in medical education by documenting the key conclusion derived from the present study: online Zoom delivery of the content in biochemistry and genetics, necessitated by the COVID-19 pandemic in 2020, yielded roughly the same student performance outcomes as on-campus classroom delivery in 2019.

## Supplementary Information

**Additional file 1.** Summary of origination site for 2019 lectures in BMB 516 and BMB 528.

**Additional file 2.** Pre-pandemic Summary of Course Grades by MSUCOM Campus Site^a^.

## Data Availability

Additional Data can be accessed via Harvard Dataverse at 10.7910/DVN/RK7KXL.

## Abbreviations

MSUCOM: Michigan State University College of Osteopathic Medicine
BMB: Biochemistry and molecular biology
EL: East Lansing
MUC: Macomb University Center
DMC: Detroit Medical Center
