# Supplementary material for: Student performance in medical biochemistry and genetics: comparing campus-based versus zoom-based lecture delivery
Source: BMC Med Educ. 2022 Nov 16;22:798. doi: 10.1186/s12909-022-03873-y (PMC9668392; doi:10.1186/s12909-022-03873-y)
Supplement: Supplementary file 1 — Additional file 1. Summary of origination site for 2019 lectures in BMB 516 and BMB 528. [file 12909_2022_3873_MOESM1_ESM.docx]

Additional file 1. Summary of origination site for 2019 lectures in BMB 516 and BMB 528

| **Date** | **Lecture Topic** | **Site of origination^a^** |
| --- | --- | --- |
| **BMB 516^b^** |  |  |
| 6/11/19 | Hemoglobin and gas transport | DMC |
| 6/11/19 | Blood pH regulation | DMC |
| 6/14/19 | Biomarkers in blood and urine | DMC |
| 6/20/19 | Overview of metabolism; carbohydrate digestion and absorption | EL |
| 6/20/19 | Oxidation states | EL |
| 6/27/19 | TCA cycle | EL |
| 7/01/19 | Storage of carbohydrates | EL |
| 7/19/19 | Fatty acid oxidation; ketone bodies; fatty acid synthesis | EL |
| 7/19/19 | Complex lipid synthesis and breakdown | EL |
| 7/26/19 | Hormones and hormone action | EL |
| **BMB 528^b^** |  |  |
| 8/26/19 | Introduction to medical genetics | EL |
| 8/27/19 | One-carbon metabolism | EL |
| 8/28/19 | Nucleotide metabolism | EL |
| 8/29/19 | DNA, nucleosomes, chromosomes | MUC |
| 9/03/19 | DNA Structure and replication | MUC |
| 9/04/19 | RNA Part 1 | DMC |
| 9/05/19 | RNA Part 2 | DMC |
| 9/10/19 | Gene expression | MUC |
| 9/11/19 | Protein translation and post-translational events | MUC |
| 9/11/19 | Cell cycle regulation | MUC |
| 9/12/19 | Stimulatory signals for proliferation | MUC |
| 9/16/19 | DNA repair | DMC |
| 9/17/19 | Factors modulating inheritance patterns | EL |
| 9/18/19 | Population genetics | MUC |
| 9/24/19 | Chromosome nomenclature and structural abnormalities | EL |
| 9/25/19 | Molecular techniques Part 1 | DMC |
| 9/26/19 | Molecular techniques Part 2 | DMC |
| 9/27/19 | Molecular techniques Part 3 | DMC |
| 9/30/19 | Common chromosomal disorders | EL |
| 9/30/19 | Micro- and macro-deletion disorders | EL |
| 10/01/19 | NF and Marfan syndrome | MUC |
| 10/01/19 | Multifactorial disorders | MUC |
| 10/10/19 | Genetic testing | DMC |
| 10/10/19 | Prenatal screening and teratogens | DMC |
| 10/11/19 | Cancer genetics | DMC |
| 10/15/19 | Clinical correlates of genetic anticipation | EL |
| 10/15/19 | Clinical correlates of genetic imprinting | EL |

**^a^** Each year, the entering class at MSUCOM consisted of ~300 students: (a) ~200 located at the East Lansing (EL) site; (b) ~50 at Detroit Medical Center (DMC) site; and (c) ~50 at Macomb University Center (MUC). Each live lecture had a point of origination (e.g. DMC) in which students were physically in the classroom with the lecturer. At the non-origination sites (e.g. EL and MUC), students watched the lecture via Polycom video conferencing in real time. There is a faculty member in each of the non-origination site classrooms who can direct questions/discussion to the lecturer or to whom students can direct questions following the session.

**^b^** The course consists of a mix of lecture and activity sessions. The activity sessions were conducted in small groups at each site. This table summarizes only those topics covered by lectures, which involved a site of origination.
