# Supplementary material for: Student performance in medical biochemistry and genetics: comparing campus-based versus zoom-based lecture delivery
Source: BMC Med Educ. 2022 Nov 16;22:798. doi: 10.1186/s12909-022-03873-y (PMC9668392; doi:10.1186/s12909-022-03873-y)
Supplement: Supplementary file 2 — Additional file 2. Pre-pandemic Summary of Course Grades by MSUCOM Campus Sitea. [file 12909_2022_3873_MOESM2_ESM.docx]

**Additional file 2. Pre-pandemic Summary of Course Grades by MSUCOM Campus Site^a^**

|  | EL | MUC | DMC | Average ± STDEV |
| --- | --- | --- | --- | --- |
| BMB 516 2018 | 87.5 | 86.1 | 87.7 | 87.1 ± 0.9 |
| BMB 516 2019 | 84.2 | 85.7 | 84.2 | 84.7 ± 0.9 |
| BMB 528 2018 | 85.5 | 85.0 | 86.5 | 85.7 ± 0.6 |
| BMB 528 2019 | 84.5 | 84.5 | 84.7 | 84.5 ± 0.5 |

**^a^** The data are expressed in terms of % of total points available in each Course. In each course, a P-grade (passing) required 70%. The last column represents the averages of the three sites for the course in that year ± the standard deviation (STDEV).
